# Should Deep-Sequenced Amplicons Become the New Gold Standard for Analyzing Malaria Drug Clinical Trials?

**DOI:** 10.1128/AAC.00437-21

**Published:** 2021-09-17

**Authors:** Sam Jones, Katherine Kay, Eva Maria Hodel, Maria Gruenberg, Anita Lerch, Ingrid Felger, Ian Hastings

**Affiliations:** a Department of Tropical Disease Biology, Liverpool School of Tropical Medicine, Liverpool, United Kingdom; b Metrum Research Group, Tariffville, Connecticut, USA; c Institute of Infection, Veterinary & Ecological Sciences, University of Liverpool, Liverpool, United Kingdom; d Medical Parasitology and Infection Biology, Swiss Tropical and Public Health Institute, Basel, Switzerland

**Keywords:** malaria, *P. falciparum*, drug trials, drug resistance, TES, molecular correction, PCR correction, *Plasmodium falciparum*

## Abstract

Regulatory clinical trials are required to ensure the continued supply and deployment of effective antimalarial drugs. Patient follow-up in such trials typically lasts several weeks, as the drugs have long half-lives and new infections often occur during this period. “Molecular correction” is therefore used to distinguish drug failures from new infections. The current WHO-recommended method for molecular correction uses length-polymorphic alleles at highly diverse loci but is inherently poor at detecting low-density clones in polyclonal infections. This likely leads to substantial underestimates of failure rates, delaying the replacement of failing drugs with potentially lethal consequences. Deep-sequenced amplicons (AmpSeq) substantially increase the detectability of low-density clones and may offer a new “gold standard” for molecular correction. Pharmacological simulation of clinical trials was used to evaluate the suitability of AmpSeq for molecular correction. We investigated the impact of factors such as the number of amplicon loci analyzed, the informatics criteria used to distinguish genotyping “noise” from real low-density signals, the local epidemiology of malaria transmission, and the potential impact of genetic signals from gametocytes. AmpSeq greatly improved molecular correction and provided accurate drug failure rate estimates. The use of 3 to 5 amplicons was sufficient, and simple, nonstatistical criteria could be used to classify recurrent infections as drug failures or new infections. These results suggest AmpSeq is strongly placed to become the new standard for molecular correction in regulatory trials, with potential extension into routine surveillance once the requisite technical support becomes established.

## INTRODUCTION

Clinical trials and therapeutic efficacy studies (TES) of antimalarial drugs are key components of public health provision in countries where malaria is endemic. The role of clinical trials is to ensure a steady supply of effective drugs, while the role of TES is to provide ongoing surveillance on the efficacy of local frontline drugs and enable rapid replacement of failing drugs to avoid the increased morbidity and mortality associated with drug resistance (1).

In principle, clinical trials and TES are simple: patients are treated with the drug being evaluated, and the number of patients with drug failures is counted to provide estimates of drug effectiveness. In practice, this is challenging because most current malaria drugs have long half-lives such that drug failures are suppressed and may only become patent several weeks after treatment. Ongoing malaria transmission means that patients often acquire new infections during the long follow-up period so a key methodological requirement of clinical trials and TES is to correctly classify patients returning with detectable malaria parasites during follow-up (termed “recurrences”) as either drug failures (termed “recrudescences”) or new infections (sometimes termed “reinfection”). This classification is achieved using molecular correction protocols, which rely on genotyping malaria parasites in the blood of the infected patient at the time of drug treatment and if that patient returns with a recurrence at any time during the 4- to 6-week follow-up period (2). “Matching” alleles between treatment and follow-up samples indicate a drug failure, while a “mismatched” allele or alleles indicate a new infection. Deciding whether or not the samples match is highly problematic, and the number of shared alleles required to define a match (and hence a drug failure) depends on the methodology used (3–5). The World Health Organization (WHO) recommends three markers (2) (i.e., the genes coding for merozoite surface protein-1 [*msp-1*], merozoite surface protein-2 [*msp-2*], and glutamate-rich protein [*glurp*]), though alternative markers are available, such as the microsatellite markers used by the Centers for Disease Control and Prevention (CDC) (6). The three WHO markers and the CDC microsatellite markers all rely on identification of alleles by their lengths (in contrast to amplicons, discussed later, where alleles differ in their sequence). Length-polymorphic genotyping uses PCR amplification of molecular markers in patient blood samples, followed by fragment sizing of the PCR products using agarose gels or capillary electrophoresis. Malaria infections often contain multiple parasite clones both at treatment (when the multiplicity of infection [MOI] is typically 3 to 8 malaria clones per person in high-transmission areas) and at recurrence (where patients may present with a mixture of recrudescence clones and new infections). There is substantial variation in the density of individual clones in these multiclonal infections, and existing methods based on length polymorphism are notoriously poor at detecting low-density clones. These methods typically regard genetic signals at less than around 20% to 30% of the major signal as “noise,” meaning any clones whose density is less than 20 to 30% of that of the dominant clone are not identified. There have been recent calls for a review of the WHO-recommended methodology, (5, 7) following scientific discussion of misclassification with length-polymorphic (*msp-1*, *msp-2*, *glurp*) (3, 7–9) and microsatellite (4, 10) approaches.

Deep sequencing of highly single nucleotide polymorphism (SNP)-polymorphic amplicons (AmpSeq) is an attractive next-generation methodology, experimentally shown to have a much greater ability to detect low-density clones than existing methods based on length-polymorphic genotyping. This has the potential to substantially improve molecular correction over existing methods. AmpSeq methods are well established in the wider malarial context for tracking specific genes (e.g., for drug resistance) within populations (11–13) and for evaluating the efficacy of the RTS,S/AS01 vaccine (14). AmpSeq as a method to genotype malaria parasites in clinical trials is relatively novel (15) and, despite its putative advantages, has not yet been used as the primary genotyping endpoint for a clinical trial or TES (although it has been used to reanalyze archived blood samples [15]). The study presented in this article used an *in silico* pharmacological approach to evaluate the putative advantages of AmpSeq for improving molecular correction. This builds on our previous work using mechanistic pharmacokinetic/pharmacodynamic (mPK/PD) modeling of malaria drug treatment to produce simulated trial data in which the “true” underlying failure rate in the simulation is known. This allowed a critical appraisal of existing TES methodology (3, 4, 16) and enabled us to quantify the accuracy of molecular correction based on length-polymorphic and microsatellite markers. A reasonable hypothesis is that the more accurate and sensitive genotyping afforded by AmpSeq will lead to more accurate efficacy estimates in clinical trials and TES than existing, widely used methods mandated by the WHO and CDC. Here, we apply this mPK/PD modeling approach to quantify the accuracy of efficacy estimates produced using published AmpSeq data of 5 SNP-polymorphic markers/loci (15). Our aim is to identify any potential problems and pitfalls *in silico* before they occur *in vivo*, optimize the likely use of AmpSeq for molecular correction in regulatory trials, and suggest appropriate guidelines for their deployment.

The main advantage of AmpSeq over existing methods is its increased sensitivity (i.e., increased ability to detect genetic signals from malaria clones present at low densities in human blood samples). Sequencing may achieve substantial read depths, but a practical problem in processing these reads is to distinguish low-number reads from artifacts. This requires a bioinformatic cutoff (BIC) value, below which low-number reads are regarded as artifacts and ignored. In the case of malaria, it appears that a BIC value of 1% is the most robust value, as extensively discussed elsewhere (17), although we also investigate a (hypothetical, perfect) BIC→0% and an alternative BIC of 2%. In all cases, the BIC should be read as the sensitivity of the method to detect low-density malaria clones: e.g., with a BIC of 1%, any malaria clone(s) whose number/density is less than 1% of the total parasitemia in a human will remain undetected.

## RESULTS

**FIG 1:**
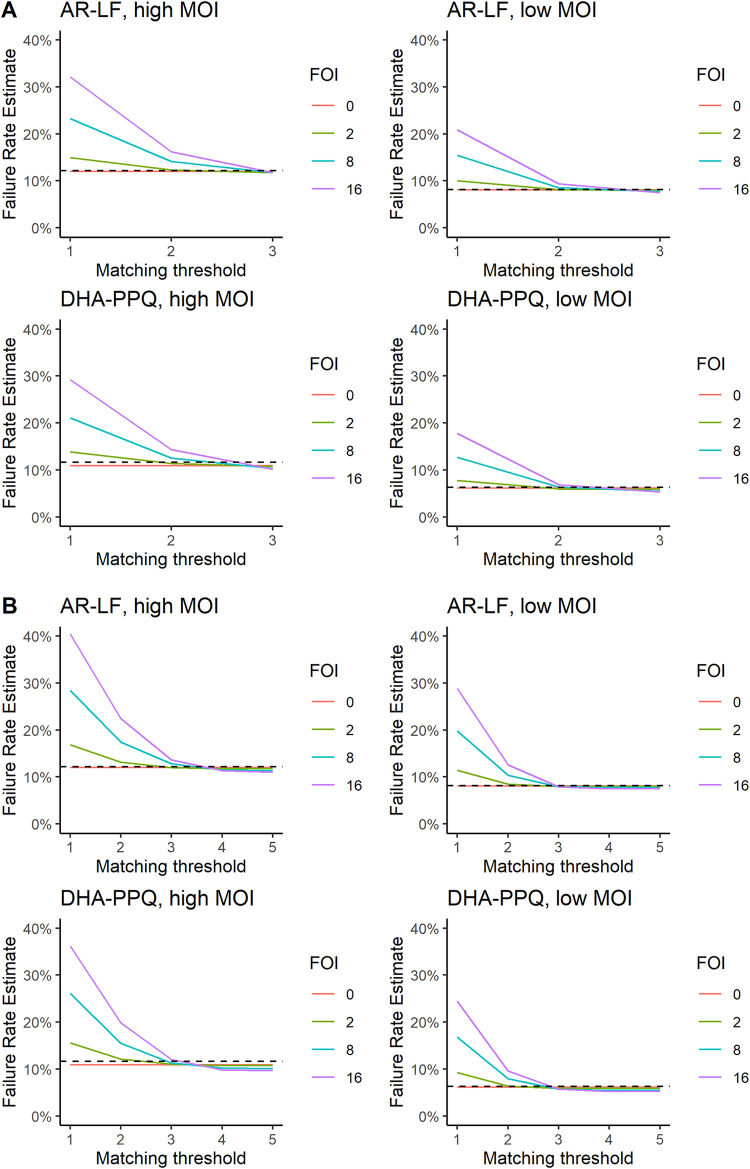
Failure rate estimates obtained using AmpSeq for dihydroartemisinin-piperaquine (DHA-PPQ) and artemether-lumefantrine (AR-LF) in settings of low and high multiplicity of infection (MOI) and a range of force of infection (FOI) values. The *x* axis shows the matching threshold used to define a drug failure (e.g., for a threshold of 2, then alleles at 2 or more loci must match in the initial and recurrent sample). The true drug failure rate is marked by the horizontal dashed black line. (A) Genotyping based on 3 AmpSeq markers. (B) Genotyping based on 5 AmpSeq markers.

Failure rate estimates for simulated populations with varied MOI and force of infection (FOI) treated with dihydroartemisinin-piperaquine (DHA-PPQ) or artemether-lumefantrine (AR-LF) are shown in Fig. 1. Three important model parameters were used as baseline scenario: BIC = 1%, a “blood sampling limit” of 10^8^ total parasites, and an initial parasite number drawn from a log-uniform distribution between 10^10^ and 10^11^. The true failure rates for DHA-PPQ were 11.6% and 6.3% in areas of high and low MOI, respectively. True failure rates increase with MOI as the drug has to successfully clear more clones present at the time of treatment; this is discussed in more detail elsewhere (16), but note that the fates of the clones are not independent because they share the same treatment “environment” within the same patient (i.e., a failure rate for MOI = 3 is not 3 times the failure rate for MOI = 1). The corresponding true failure rates for AR-LF were 12.2 and 8.2%. Failure rate depends on MOI for the reasons given above. However, it does not depend on FOI because FOI only determines the reinfection rate. We count a drug failure as anyone whose treatment fails to clear all of the parasites present at treatment, irrespective of whether or not the recrudescence is masked by a new infection.

A matching threshold of ≥2 or 3 AmpSeq loci used to classify recurrent infection as recrudescence produced highly accurate failure rate estimates when analyzing 3 loci (Fig. 1A) or using thresholds of ≥3, ≥4, or 5 when analyzing five loci (Fig. 1B). Molecular correction using Ampseq therefore performed much better than previously observed in our analysis of similar matching thresholds based on length-polymorphic WHO-recommended markers and for microsatellites (3, 4). This was true under all scenarios: i.e., for DHA-PPQ and AR-LF in both high and low MOI and across all FOI values (see supplemental material, part 2). The accuracy of using three loci implies it is unnecessary to genotype more AmpSeq loci, and this appears to be the case: genotyping 4 or 5 additional loci did not improve accuracy (Fig. 1B; see supplemental material, part 2).

An important operational question is whether technological advances capable of reducing BIC to below 1% will result in better estimates. This is unlikely given the accuracy of using BIC = 1% (i.e., Fig. 1), but we reran the analyses using the theoretical minimum value of BIC→0% and, as expected, found no improvement. Notably, increasing BIC to 2% also had a negligible impact on accuracy (supplemental material, part 2).

Sensitivity analyses were conducted by repeating simulations with altered model parameters to confirm their values did not affect the conclusions. A lower blood sampling limit or increasing initial parasite distributions showed no qualitative differences and negligible quantitative differences from the results shown in Fig. 1 (supplemental material, part 2).

## DISCUSSION

Existing research has identified suitable SNP-polymorphic AmpSeq loci for genotyping malaria parasites (14, 17) and confirmed and quantified their superior ability to detect low-density clones compared to traditional length-polymorphic genotyping methods (15, 17, 18). AmpSeq also provided improved estimates of MOI (19) and identified appropriate thresholds for allele detection (17, 20). Here, we aimed to quantify the hypothesized increase in the accuracy of failure rate estimates in clinical trials (and eventually TES) that should result from AmpSeq’s increased ability to detect low-density clones.

Figure 1 (and see Fig. S2.5 in the supplemental material, which shows analogous results for 4 loci) suggests an important diagnostic in the use of AmpSeq that serves (i) to check that molecular correction is based on a sufficient number of AmpSeq loci and (ii) to identify an appropriate choice of matching threshold that enables AmpSeq markers to solidly distinguish recrudescences from new infections. At the lowest threshold of 1, unrelated parasite clones of the sample pair (i.e., treatment and recurrence) may match purely by chance (often due to a dominant allele), meaning that a new infection would be mistakenly classified as recrudescence (8). As the threshold increases, this probability of matching by chance declines to negligible levels, and failure estimates become stable with respect to threshold. Providing this pattern of rapid fall to a plateau occurs, the choice of matching threshold can be any that lie on the “flat” part of the curve. Our plots suggest we could use matches at 2 or 3 loci when using 3 Ampseq markers (Fig. 1A), 3 or 4 matches when using four markers (Fig. S2.5), and 3, 4, or 5 matches when using 5 markers (Fig. 1B). Note that the appropriate choice of threshold depends on the study site because the probability of matching by chance increases if the AmpSeq markers are less diverse than those simulated here and/or as transmission intensity (FOI) increases the genetic complexity of the infections (MOI). Rather than recommending a universal threshold *a priori*, we recommend that the choice be based on the diagnostic plot generated for each study (i.e., demonstration that the plot flattens and threshold occurs in the flat portion), so this diagnostic is likely to become essential to validate the methodology as clinical data start to accumulate. It was notable that addition of two less diverse markers to our core 3 AmpSeq markers still passed this diagnostic (Fig. 1B), suggesting only a few, well-characterized AmpSeq loci may be required to achieve accurate molecular correction.

This diagnostic is one reason why we regard AmpSeq as a the potential new “gold standard.” Genotyping based on *msp-1*/*msp-2*/*glurp* fails this diagnostic because the curve continues to fall and never reaches a plateau. Use of *msp-1*/*msp-2*/*glurp* appears to provide accurate overall failure rate estimates, provided a recrudescence is defined in a ≥2/3 algorithm (3). However, it is important to note that classification of individual patients is often incorrect in the ≥2/3 algorithm applied to *msp-1*/*msp-2*/*glurp*, but the errors balance (i.e., the number of recrudescences misclassified as new infections is roughly equal to the number of new infections misclassified as recrudescences; see Fig. 3 in reference 3). This is obviously rather unsatisfactory because the balance can be shifted by factors such FOI and duration of follow-up. AmpSeq seems to provide accurate classification of individual patient outcomes, which is much preferable to balancing errors and allows more accurate correlations between treatment outcome and underlying risk factors: i.e., it allows the presence of drug resistance mutations to be more closely tested against individual treatment success/failure. Note that this correlation is usually obtained as an odds ratio for the presence of a drug resistance marker at treatment and patient failure (21). There is currently no way of achieving the logical next step (i.e., to construct the clonal haplotype that contains both the drug marker and its Ampseq markers) so that the odds ratio of a resistance clone failing treatment could be calculated (although, in principle, this may be possible if the MOI is low and the clones differ substantially in their density such that haplotypes could be inferred).

The results presented here assume alleles can only be detected at frequencies of >1% within a sample (i.e., BIC = 1%). In reality, experimental mixtures suggest that AmpSeq is potentially even more sensitive than this, but BIC = 1% is required to avoid inclusion of PCR errors/artifacts and environmental contaminations (15). A BIC value of 1% reflects present technology for robust genotyping (15), but we wished to anticipate and evaluate technological advances that may reduce this limit. Our results show that reducing BIC to the hypothetical perfect detection limit as BIC→0% (and assuming no false positive occurred) *in silico* made a negligible difference to the accuracy of the method; this most likely occurs because low-density clones that could be detected by the hypothetical perfect BIC→0% are likely to be below the blood sampling limit (i.e., are unlikely to physically enter the finger prick samples used in clinical trials and routine TES). The additional results (supplemental material, part 2) also showed that BIC could be increased to 2% with negligible reductions in accuracy of molecular correction. Many surveys have shown a high prevalence of extremely-low-density clones by deep sequencing venous blood samples whose volumes are several magnitudes larger than that of a finger prick. Whether resistant clones present at such extreme low density are likely to recrudesce after treatment is unknown. (One argument is that they are controlled by host immunity and so are unlikely to recrudesce.) Unfortunately, we cannot genotype such low-density clones and test this directly in field trials using current technology because, as described above, the sequence depth is not the issue; any amplification step in the genotyping protocol will limit the sensitivity to around BIC = 1% to avoid contamination producing too many low-density false-positive haplotype calls. Furthermore, the setting BIC = 1% reduces the risk of detecting genotypes of gametocytes persisting from treatment; the dangers of this have been raised previously (e.g., references 15 and 22) and are quantified in part 3 of our supplemental material.

**FIG 2:**
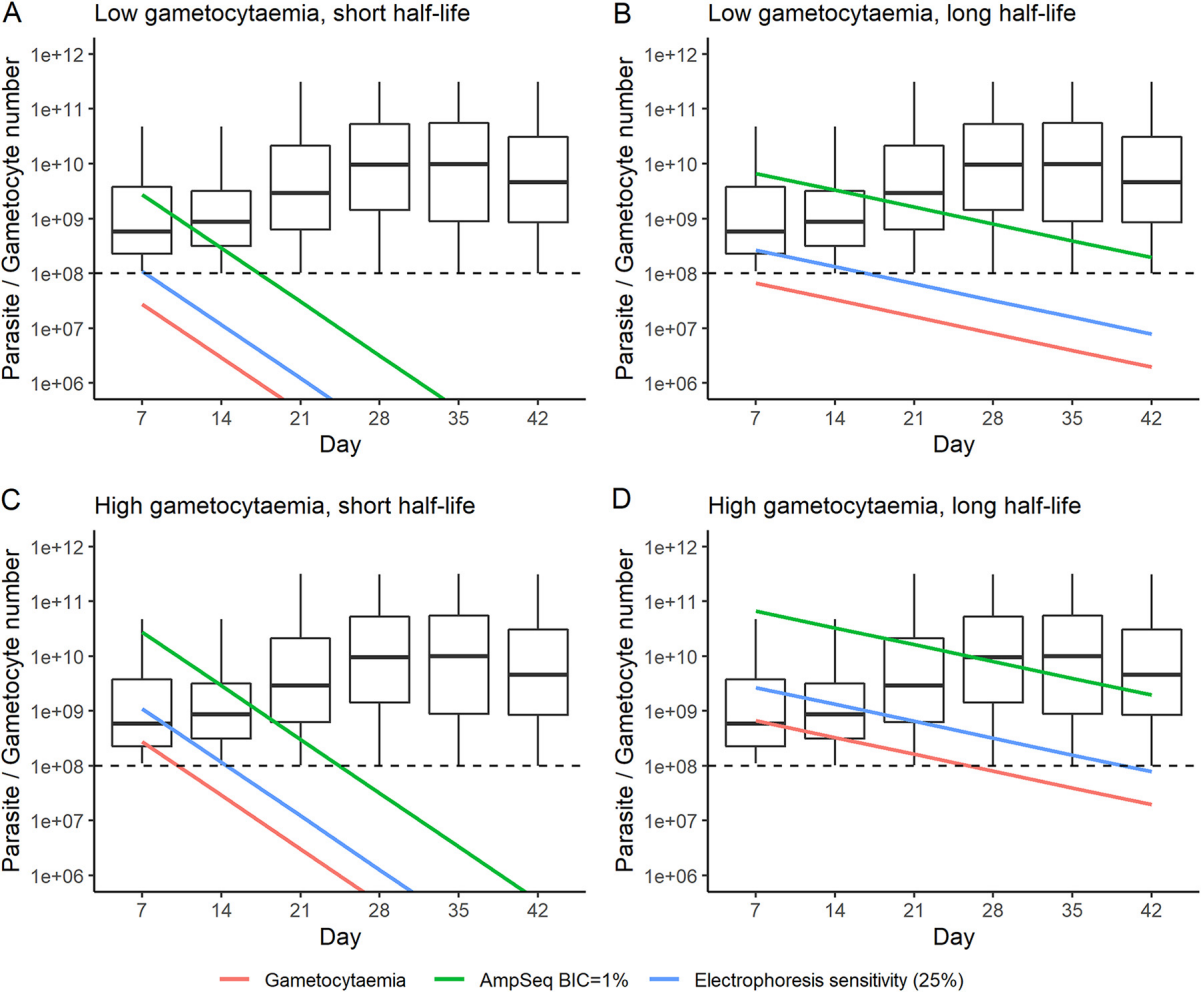
The potential impact of gametocyte genetic signals on molecular correction. The box plots show total asexual parasitemia at the time of recurrence for 5,000 patients with new infections treated with DHA-PPQ (with early treatment failures on day 3 excluded). The potential impact of gametocyte genetic signals is demonstrated by modeling the gametocytemias posttreatment of four illustrative gametocytemia clones present at treatment. The red lines show gametocyte number. The green lines are 100× gametocyte number: since we are assuming BIC = 1%, new infections in the box plots whose asexual parasite number lies below these green lines will potentially have alleles from these gametocytes detectable when using AmpSeq. The blue lines are 4× gametocytemia: standard WHO genotyping based on gel-electrophoresis has a sensitivity to detect “minor” genetic signals down to around 25% of the total parasitemia, so new infections in the box plots whose asexual parasite number lies below these blue lines will potentially have alleles from these gametocytes detectable using the standard WHO methodology. The horizontal dotted line at 10^8^ is the blood sampling limit (i.e., total number of gametocytes in the patient): when gametocytemia falls below this level, gametocytes are highly unlikely to physically enter a standard finger prick blood sample and so will remain undetected. Note that the *y* axis is calibrated as the total number of parasites/gametocytes in the infection because this is the parameter tracked in the standard PK/PD modeling methodology; for conversion to parasite densities, see the discussion in section 1.5 in the supplemental material. (A) An illustrative clone with 10^8^ gametocytes at the time of treatment and a gametocyte half-life of 2.15 days. (B) An illustrative clone with 10^8^ gametocytes at the time of treatment and a gametocyte half-life of 6.86 days. (C) An illustrative clone with 10^9^ gametocytes at the time of treatment and a gametocyte half-life of 2.15 days. (D) An illustrative clone with 10^9^ gametocytes at the time of treatment and a gametocyte half-life of 6.86 days.

The possibility of detecting gametocyte signals when genotyping blood samples in follow-up is illustrated in Fig. 2, whose panels should be interpreted as follows: provided gametocytemia is above the blood sampling limit (i.e., >10^8^ gametocytes in the human host as shown by the horizontal dotted line) at time of recurrence, then each of the four exemplar clones will be:

• Detectable by Ampseq in all recurrences in the box plots whose parasitemia lies below the green line (because gametocytes in that clone are present at >1% of total parasitemia).
• Detectable by standard length polymorphism (e.g., the standard WHO-recommended methods based on *msp-1*, *msp-2*, and *glurp*) in all recurrences in the box plots whose parasitemias lie below the blue line (because gametocytes in that clone are present at >25% of total parasitemia).

Malaria clones with “low” gametocytemia at treatment (which is actually still rather high) are likely to have fallen below the blood sampling limit by the first day of follow-up (day 7) and so are highly unlikely to be detected when genotyping recurrences irrespective of whether the gametocytes have long or short half-lives (Fig. 2A and C). Malaria clones with high gametocytemia at treatment do have the potential to be detected during follow-up. Gametocytes with short circulatory half-life may still be above the blood sampling limit on day 7 (Fig. 2C), and the clone would be detectable on that day in almost all recurrences genotyped by AmpSeq (green line) and in most recurrences when genotyped by length polymorphism. Gametocytes with a long circulatory half-life remain above the blood sampling limit for up to 21 days (Fig. 2D), during which time they will be detectable by AmpSeq in almost all recurrences (green line) and often detectable by length-polymorphism genotyping (blue line). Figure 2 shows the detectability of 4 exemplar clones. The overall impact of persisting gametocytes on molecular correction will depend on how frequently these different types of clones are present in the trial study site. Studies carried out in low-gametocytemia patients will almost certainly not be affected by detection of gametocyte signals, while those enrolling patients with high gametocytemias may have subsequent molecular correction compromised through detection of genetic signals from gametocytes during follow up, particularly if gametocytes have moderate to long half-lives (see also some further discussion in the supplemental material, part 3).

The results presented above emphasize that the quality rather than quantity of amplicon loci is the key to obtaining accurate drug failure rates. Relatively large suites of amplicon markers (often 100+) are being developed for population genetic analyses of species, including *Plasmodium* spp., but these require strict quality control before being used for molecular correction. Based on our previous validation of this methodology, strict control of sequencing errors was applied (15): i.e., taking three independent replicates per dried blood spot sample and only classing an allele as present if (i) it was detected in all three replicates at more than 1% of total reads (i.e., BIC = 1%) and (ii) there were a minimum of 10 reads per allele and 50 reads per amplicon in each replicate. No compromise should be acceptable regarding data quality and efforts made to eliminate sequencing errors. Use of an optimized technique, such as performing all molecular analyses in triplicate, applies to genotyping in regulatory clinical trials as well as surveillance. Thus, high costs represent a serious limitation. The final check before using amplicons for molecular correction is to confirm there is sufficient local genetic diversity (quantified as expected heterozygosity) (Table 1) at the amplicon to ensure that there is only a small probability of two independent blood samples sharing the alleles purely by chance. A limitation could consist of few dominant haplotypes of a marker that together account for 50% allelic frequency. In high-transmission areas with high MOI, such a marker could cause misclassification and bias toward recrudescence (15). In our simulation, assuming high levels of genetic diversity, using more than around 5 amplicons resulted in little, if any, improved accuracy (and may even increase the risk of genotyping errors).

**TABLE 1:** Summary of parameters used in the simulation and their values^*a*^

Parameter	Description and/or values in simulations
Drugs simulated (ACT)	DHA-PPQ and AR-LF
Initial parasitemia	No. of individual parasites in a clone present at treatment: log-uniform distributions of 10^10^ to 10^11^ (default) or 10^8^ to 10^11^ (for sensitivity analysis)
Blood sampling limit	No. of parasites in a clone that must be present to ensure detection via finger prick blood sampling: 10^8^ (default) or 10^7^
MOI	No. of detectable malaria clones in a person at treatment; “high” MOI, mean = 3.6 with values of 1–8 at frequencies of 0.036, 0.402, 0.110, 0.110, 0.183, 0.049, 0.061, and 0.049, respectively; “low” MOI, mean = 1.7 with values of 1–4 at frequencies of 0.460, 0.370, 0.150, and 0.020, respectively
FOI	No. of new infections/person/yr: 0, 2, 8, or 16
Patient sampling during follow-up	Day 3, day 7, then every 7 days thereafter up to day 28 for AR-LF and day 42 for DHA-PPQ
Amplicon loci	*cpmp* (He = 1.0), *ama1-D3* (He = 0.98), *cpp* (He = 1.0), *csp* (He = 0.97), *msp-7* (He = 0.91)
BIC	Percentage of total reads an amplicon must exceed to confirm its presence in patient blood sample: 1% (default), 2%, or ->0%
Gametocytes	
Initial no.	Initial no. present at treatment: 10^8^ or 10^9^
Lag period until drug-induced decline in gametocyte no.	Dependent on drug activity against mature and/or maturing gametocytes: assumed to be 3 days for the ACTs investigated here
Half-life	Rate at which gametocyte no. falls after the lag period has ended: short at 2.15 days or long at 6.86 days

a ACT, artemisinin combination therapy; DHA-PPQ, dihydroartemisinin-piperaquine; AR-LF, artemether-lumefantrine; MOI, multiplicity of infection; FOI, force of infection; He, expected heterozygosity (taken from Table 1 of reference 15); BIC, bioinformatic cutoff.

Traditional molecular correction with length-polymorphic markers has been conducted using either gel-based or capillary electrophoresis (CE) of PCR products. The 2007 WHO guidelines contained protocols for both gel-based electrophoresis and CE (2, 9), but CE-based sizing using an automated sequencer offers higher sensitivity and ability to discriminate between alleles with minimal size differences; CE is now widely used and has generally phased out gel-based electrophoresis for molecular correction (9, 17). However, this improved discriminatory power is unable to compensate for the key deficiency of such methods (i.e., the inability to detect genetic signals from low-density clones), and our results clearly demonstrate the superiority of using amplicons for molecular correction. The adoption of AmpSeq will require use of next-generation sequencing platforms. The economic cost of reagents and deploying these machines to sub-Saharan Africa and Southeast Asia for use in malaria surveillance is likely to be significant and will necessarily need substantial bioinformatics expertise and special training for sequencing data analysis with existing software (e.g., HaplotypR, SeekDeep, and PASEC) (17, 20, 23), reagent supply, and equipment maintenance. Having AmpSeq facilities in every sentinel site is unlikely to be feasible, particularly in the short term. The future putative deployment of AmpSeq for analysis of routine malaria TES would probably require equipping a central site—one per country or even regionally if necessary—with the technology and expertise required to implement the methodology. Economic and technological factors appear to be the largest obstacle for AmpSeq deployment as a molecular correction methodology in routine TES surveillance but should be balanced against the long-term economic benefits of generating the accurate failure rate estimates required to ensure a sustainable supply of effective antimalarial drugs.

Finally, we stress that we have presented *in silico* simulations that we believe are a necessary prerequisite for understanding how AmpSeq genotyping is likely to behave during analysis in real clinical trials or TES for molecular correction. There is no guarantee that what occurs *in silico* will fully reflect what occurs in practice, but our results strongly suggest that simple analysis of three to five genetically diverse Ampseq loci should provide a baseline platform to obtain accurate drug failure rate estimates in trials and TES. This will need to be tested in early trial analysis. For example, researchers may choose to genotype a larger bank of AmpSeq markers, in which case our results suggest choosing the best (i.e., reliably genotyped, genetically diverse loci) three to five loci should be sufficient to obtain good failure rate estimates. Similarly, genotyping technology may improve such that BIC may routinely fall below 1%. Analyses may improve by incorporating the probability that a recurrence can match the treatment genotype purely by chance (which is low for the genetically diverse AmpSeq we simulate). Finally, designing a Bayesian analysis of AmpSeq genotyping (as previously developed for microsatellites in Plasmodium falciparum [10] and similar outcomes such as recurrences in Plasmodium vivax [24]) may be more accurate than simply counting the number of matches. It remains to be seen whether these improvements will have a significant, or a negligible, impact on failure rate estimates. One danger of more sophisticated analyses is that researchers often do not engage with the processes: they either ignore them in favor of simpler methods, or the implementation is so complicated that misunderstandings arise. For example, a recent review (25) showed that many TES performed in sub-Saharan Africa did not conform to current WHO guidelines for classifying recurrences as drug failures or new infections based on simple identification of shared alleles. Subsequent analysis frequently regarded new infections as equivalent to drug cure (25), again contrary to WHO guidelines that recommend their removal from the analysis or incorporation into survival analysis. It is important to recognize that in-country researchers generating genotyping data naturally wish to analyze their own data, rather than passing it to external collaborators, which is why simple counting is operationally preferable to the slightly more accurate Bayesian analyses that are likely to be technically inaccessible to most groups.

In summary, the results presented here suggest AmpSeq is a strong candidate to become the new gold standard method of molecular correction in malaria clinical trials as such studies would be supported by the requisite technical expertise. As infrastructure and technical expertise increases in countries where malaria is endemic, the use of amplicons may eventually become feasible in routine TES. Simulations suggest that accuracy would not improve significantly from the current methodology even if a hypothetical perfect detection of low-density clones was achievable (i.e., BIC→0%), implying that AmpSeq is unlikely to be usurped by newer, more sensitive genotyping technologies. In fact, further increasing sensitivity (i.e., reducing BIC) may potentially become counterproductive through capturing false-positive signals from gametocytes (supplemental material, part 3). Analyses of AmpSeq data are also straightforward and can use simple counting of allelic matches to distinguish recrudescence from new infection.

## MATERIALS AND METHODS

### Simulating parasitemias and their genotypes in therapeutic efficacy trials.

Published mPK/PD methodologies (3, 4) were used to generate data sets of parasite numbers over time posttreatment for 5,000 simulated adult patients treated with either dihydroartemisinin-piperaquine (DHA-PPQ) or artemether-lumefantrine (AR-LF). These simulated patients differed in key factors related to their parasite dynamics posttreatment: i.e., their individually assigned PK parameters, the level of resistance in the patient’s parasites (the PD element), their parasitemias at treatment, the local intensity of transmission, and so on, as discussed above. Note that mPK/PD methodology tracks number, rather than density, of parasites (see supplemental material, part 1, for how to interconvert these metrics). The simulations produce parasitemia posttreatment for each patient simulated in the trial. Specifically, we track individual parasite clones present at treatment (which may be cleared or recrudesce) and new infections.

These simulated patients were followed up for 42 days following DHA-PPQ treatment or 28 days following AR-LF treatment and “tested” for recurrent parasitemia by light microscopy on the scheduled days of follow-up: i.e., days 3 and 7 and every week thereafter in line with WHO guidelines (26). For each patient, our model calculated the day of follow-up when recurrent parasites were first detectable according to the procedure described in reference 3, noting that some patients will never show recurrence for one of two reasons: i.e., (i) the drug cleared the original infection and the patient never acquired a patent new infection during follow-up, or (ii) the drug did not clear the original infection, but follow-up ended before the recrudescence became patent.

Genetic diversity of infections at treatment is termed the multiplicity of infection (MOI), which is the number of (detectable) genetically distinct parasite clones in the blood sample. MOI depends on intensity of transmission, so “high” and “low” MOIs were explored. Each parasite clone within the MOI had a total number of parasites drawn from a log-uniform distribution according to two ranges: 10^10^ to 10^11^ (the default range) and 10^8^ to 10^11^ (for sensitivity analysis). Genetic diversity in recurrences depends on the force of infection (FOI), which is the rate at which new infections become established in a patient. We incorporated FOI as the mean of a Poisson distribution from which the days of new infections are randomly selected for each patient. The FOI means were 0, 2, 8, and 16 per year, broadly representing areas with no, low, medium, and high ongoing transmission, respectively. New infections emerge from the liver as cohorts of 10^5^ total parasites, and their fate (to be cleared or to survive) depends on drug concentrations in that host on the days following their emergence from the liver. (Many emergences that appear shortly after treatment will be killed by the highly persistent partner drug, PPQ or LF.) Each malaria clone at treatment or recurrence had genotypes defined at five AmpSeq markers (*cpmp*, *ama1-D3*, *cpp*, *csp*, and *msp-7*) with allelic diversity obtained from reference 15. We used the first three markers by default but also included the less diverse markers *csp* and *msp-7* in our simulations as these may be used when other markers fail to amplify (15), and this allows us to evaluate whether increasing the number of AmpSeq markers would significantly improve accuracy.

A technical description of this methodology, including mPK/PD parameters and references to previous work, is provided in the supplemental material, part 1. All simulations were conducted using the programming language R (version 1.2.5001) (27). The simulated data from the trial were then processed as follows.

### Allele detection in simulated data sets: the blood sampling limit and bioinformatic cutoff.

Our model determined which AmpSeq alleles were detected in initial and recurrent samples with a modified version of the methodology described in references 3 and 4 and in the supplemental material, part1. It follows a two-stage process as follows:

1. A clone must have a sufficiently high parasitemia that infected erythrocytes physically enter a finger prick blood sample; this is obviously a prerequisite (but not a guarantee) for their later detection by PCR. This limiting level of parasitemia is termed the “blood sampling limit” (3, 4), and two limits were utilized: 10^8^ total parasites, as used previously (3), and 10^7^ total parasites.
2. An amplicon allele will only be identified by the bioinformatics pipeline as present in the blood sample if it exceeds an empirically determined threshold number and/or proportion of total reads. (If below this threshold, the reads are regarded as “noise.”) We call this threshold the bioinformatic cutoff (BIC) inherent in the experimental protocol. In our previous work (15), only reads present at >1% were regarded as “real” as this is highly robust (15); consequently, we use this value (i.e., BIC = 1%) as the baseline for our simulations. We also investigated a more stringent threshold of 2% (i.e., BIC = 2%). Since BIC is experimentally determined, we must anticipate settings where, for example, researchers are sufficiently confident in their technology and results that they regard reads present at proportions above 1 in 500 of the total reads or even above 1 in 1,000 (i.e., BIC = 0.02% or BIC = 0.1%, respectively) as confirming the presence of that AmpSeq allele. Also, future technical advances may allow researchers to reduce this threshold even further: e.g., to BIC = 0.001%. Rather than investigate all of these BIC thresholds separately (e.g., BIC = 0.2%, 0.1%, 0.001%, etc.), we take the approach of investigating how the results would change as the threshold became extremely low: i.e., BIC = 0.0000 … 1% (i.e., as BIC tended to zero [BIC→0%]). The logic is that if there is little improvement in accuracy as BIC falls from BIC = 1% to BIC→0, then other values (such as the exemplar values BIC = 0.2%, 0.1%, and 0.001%) would also have little impact.

### Matching threshold for molecular correction and subsequent estimation of failure rate.

Classification of recurrences as drug failures (i.e., recrudescences) or new infections was primarily based on the three most diverse markers, *cpmp*, *cpp*, and *ama1-D3*. A “matching threshold” is used to classify a recurrence as a recrudescence when the number of markers with at least one shared allele between the initial and recurrent samples was greater than or equal to the matching threshold. For example, a threshold of ≥2 meant the initial and recurrent samples must share alleles at 2 or more markers to classify the recurrence as recrudescence. Recurrences below this threshold were classified as new infections. Once all recurrences are classified as recrudescences or new infections, the drug failure rate estimates in the trial were calculated using survival analysis, as per the WHO procedure (26).

### The potential impact of gametocyte genotyping signals.

Mature falciparum infections often contain relatively high densities (up to 10% of total parasitemia) of their transmission stage, gametocytes. These stages do not cause symptoms, are unaffected by most drugs, and decline slowly posttreatment, with a half-life of around 2 to 6 days. This slow decline has raised concerns that their genetic signals could be detected when genotyping recurrences using highly sensitive genotyping techniques such as AmpSeq. These signals would be mistaken for signals arising from persisting asexual stages, would be counted in the matching threshold described above, and hence bias molecular correction by increasing the likelihood of a “match” between original and recurrent infections (i.e., will potentially generate “false-positive” recrudescences). Assuming we know the number of gametocytes at treatment, the lag time before they start to decline, and the rate of decline thereafter, it is straightforward to track their numbers following treatment and hence their potential detection and impact on molecular correction. However, discussions of calibration, algebra, and presentation of results became rather lengthy so, to maintain focus in the main text, we describe how we simulate their likely impact in a stand-alone section of the supplemental material, part 3.

We provide a summary of the parameters used in the simulations and their values in Table 1.
